# STAT3 regulates glycolysis via targeting hexokinase 2 in hepatocellular carcinoma cells

**DOI:** 10.18632/oncotarget.15801

**Published:** 2017-03-01

**Authors:** Man Li, Rui Jin, Weihua Wang, Tieying Zhang, Jiao Sang, Na Li, Qunying Han, Wenxuan Zhao, Chunyan Li, Zhengwen Liu

**Affiliations:** ^1^ Department of Infectious Diseases, First Affiliated Hospital of Xi'an Jiaotong University, Xi'an 710061, Shaanxi, People's Republic of China; ^2^ Department of Internal Medicine, The Third Hospital of Xi'an, Xi'an 710021, Shaanxi, People's Republic of China; ^3^ Department of Radiology, Second Affiliated Hospital of Xi'an Jiaotong University, Xi'an 710004, Shaanxi, People's Republic of China; ^4^ Department of Pharmacogenomics, The Fourth Military Medical University, Xi'an 710032, Shaanxi, People's Republic of China

**Keywords:** hepatocellular carcinoma, signal transducer and activator of transcription 3, hexokinase 2, glycolysis, rapamycin

## Abstract

Signal transducer and activator of transcription 3 (STAT3) and hexokinase 2 (HK2) are involved in hepatocellular carcinoma (HCC). Deregulation of cellular energetics involving an increase in glycolysis is a characteristic of HCC. This study examined whether STAT3 regulates HCC glycolysis through the HK2 pathway in HCC cells. Human HCC cell lines HepG2 and Hep3B cells were transfected with pcDNA3.1(+)-EGFP-STAT3, STAT3 siRNA and HK2 siRNA, respectively, or treated with rapamycin, an inhibitor of mammalian target of rapamycin (mTOR), and the effects on STAT3 and HK2 expression and cell glycolysis were determined. STAT3 and HK2 expressions were evaluated by real-time polymerase chain reaction and Western blotting. The level of glycolysis metabolism was assessed by the determination of glucose consumption and lactate production.The results showed that transfection of HepG2 and Hep3B cells with pcDNA3.1(+)-EGFP-STAT3 significantly increased STAT3 mRNA and protein expression, glucose consumption and lactate production, and HK2 mRNA and protein expression. However, transfection of HepG2 and Hep3B cells with STAT3 siRNA significantly decreased glucose consumption and lactate production and HK2 mRNA and protein expression. Transfection of HepG2 and Hep3B cells with HK2 siRNA significantly decreased glucose consumption and lactate production. Treatment of HepG2 and Hep3B cells with rapamycin significantly reduced HK2 mRNA and protein expression and glucose consumption and lactate production. These results suggest that mTOR-STAT3-HK2 pathway is involved in the glycolysis of HCC cells and STAT3 may regulate HCC glycolysis through HK2 pathway, providing potential multiple therapeutic targets through intervention of glycolysis for the treatment of HCC.

## INTRODUCTION

Cancer cells preferentially use aerobic glucolysis to metabolize glucose [1, 2]. High glycolysis, a phenomenon known as the “Warburg effect” [3], is often observed in various cancers [4, 5]. The first step of aerobic glycolysis is catalyzed by enzyme hexokinase [6]. Hexokinase 2 (HK2), an isoform of hexokinase, catalyzes the first irreversible step of the glycolysis and helps couple ATP formation in mitochondria to glucose phosphorylation, resulting in cancer cell growth, survival and metastasis [4–10].

Signal transducer and activator of transcription 3 (STAT3) signalling is a major pathway in cancer initiation and malignant progression [11–13]. STAT3 has been revealed to potentiate glucose metabolism and accelerate glycolysis by upregulating HK2 in breast, bladder, and ovarian cancer cells [14–18].

HK2 expression in hepatocellular carcinoma (HCC) has been shown to be associated with tumor grade and stage as well as increased mortality of HCC, suggesting the biologic and prognostic significance of HK2 in HCC [19, 20]. Overexpression of HK2 in HCC cells has been shown to be associated with increased tumor cell survival and proliferation and resistance to the anticancer agent cisplatin [21]. Furthermore, HK2 expression has been indicated to be significantly correlated with STAT3 expression in hepatitis B virus (HBV)-related HCC [22]. Deregulation of cellular energetics involving an increase in glycolysis is also a characteristic of HCC [23, 24]. However, whether the HCC glycolysis is attributable to STAT3 and whether HK2 pathway is correlated to this process remain largely unknown. Therefore, this study investigated the potential mechanism of STAT3 attribution to HCC glycolysis through regulating HK2 in HCC cells.

## RESULTS

### Effect of STAT3 on glycolysis in HepG2 and Hep3B cells

**Figure 1:**
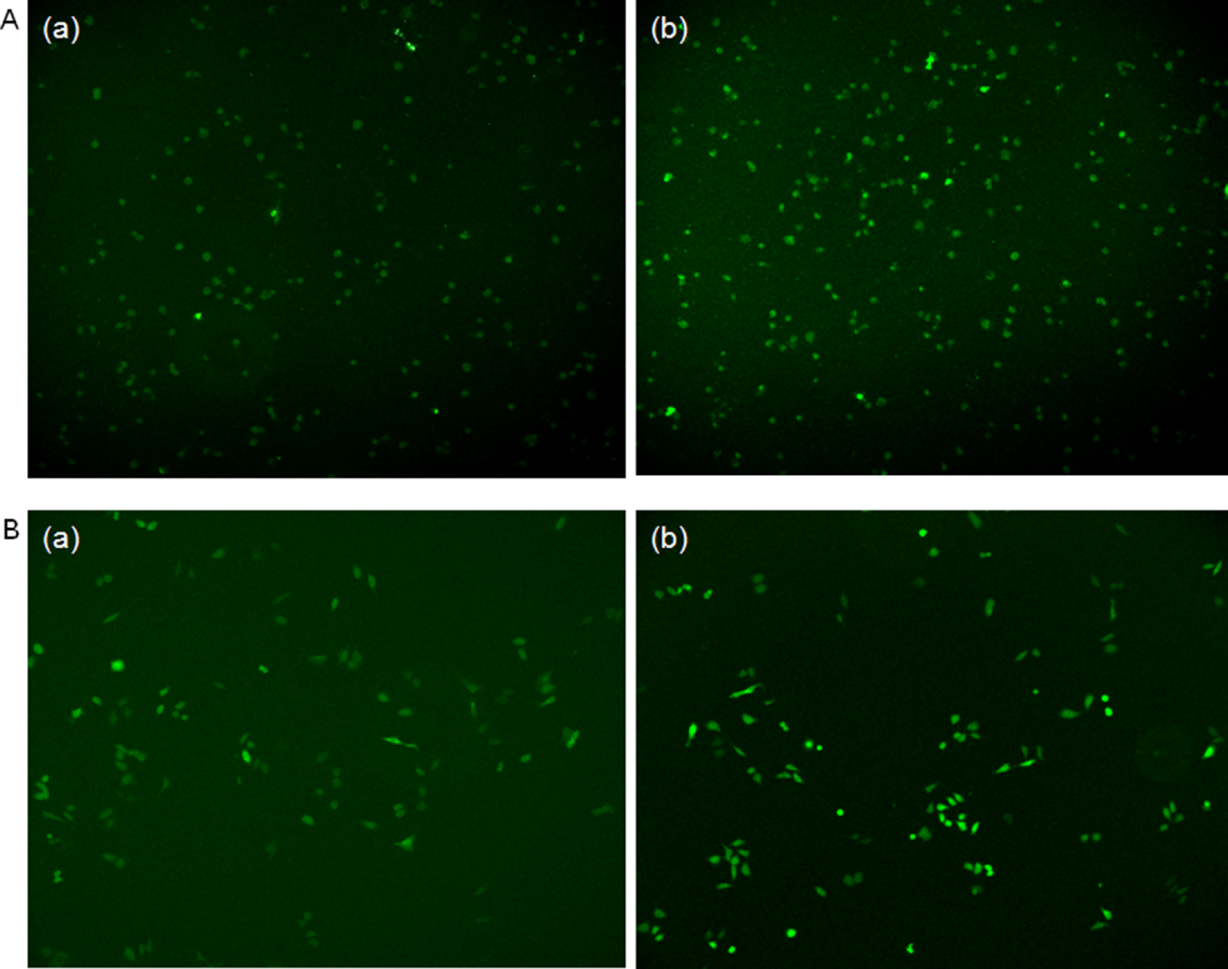
Immunofluorescent staining of STAT3 expression in HepG2 and Hep3B cells transfected with pcDNA3.1-STAT3 in comparison with pcDNA3.1-Mock plasmids (**A**), (a) HepG2 cells transfected with pcDNA3.1-Mock plasmid for 48 h. (b) HepG2 cells transfected with pcDNA3.1-STAT3 plasmid for 48 h. (**B**) (a) Hep3B cells transfected with pcDNA3.1-Mock plasmid for 48 h. (b) Hep3B cells transfected with pcDNA3.1-STAT3 plasmid for 48 h.

The plasmids pcDNA3.1-STAT3 (pcDNA3.1-S) and pcDNA3.1-Mock (pcDNA3.1-M) were successfully constructed. DNA sequencing confirmed the correctness of sequence of the constructs. The Immunofluorescent staining results showed significant increase of STAT3 expression in HepG2 and Hep3B cells transfected with pcDNA3.1-STAT3 in comparison with pcDNA3.1-Mock (Figure 1).

**Figure 2:**
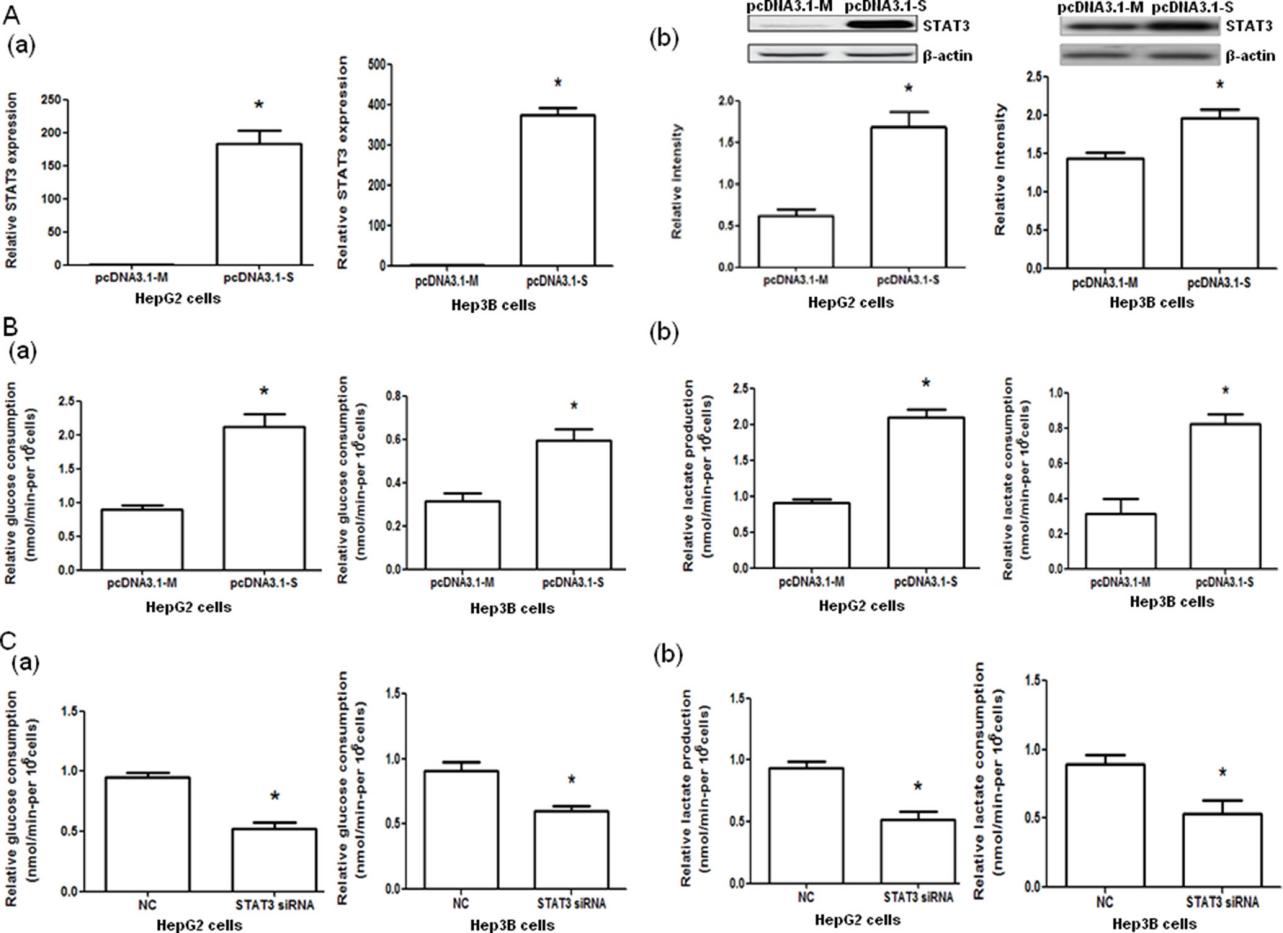
STAT3 mRNA and protein expressions and glucose consumption and lactate production in HepG2 and Hep3B cells with STAT3 overexpression and interference (**A**) The STAT3 mRNA (a) and protein (b) expression in HepG2 and Hep3B cells transfected with control pcDNA3.1-Mock (pcDNA3.1-M) and pcDNA3.1-STAT3 (pcDNA3.1-S) determined by quantitative PCR and Western blot analysis, respectively. (**B**) The glucose consumption (a) and lactate production (b) of HepG2 and Hep3B cells transfected with control (pcDNA3.1-M) or pcDNA3.1-STAT3 (pcDNA3.1-S). (**C**) The glucose consumption (a) and lactate production (b) in HepG2 and Hep3B cells transfected with negative control (NC) RNA and STAT3 siRNA. *P < 0.05.

HepG2 and Hep3B cells transfected with pcDNA3.1-STAT3 (pcDNA3.1-S) showed significantly increased STAT3 mRNA and protein expressions (both *P <* 0.05, Figure 2A) and significantly elevated glucose consumption and lactate production (both *P <* 0.05, Figure 2B) compared with cells transfected with pcDNA3.1-Mock (pcDNA3.1-M). Furthermore, the transfection of HepG2 and Hep3B cells with STAT3 siRNA significantly decreased the glucose consumption and lactate production in HepG2 and Hep3B cells compared with negative controls (both *P <* 0.05, Figure 2C, Supplementary Table 1).

### Effect of STAT3 on hexokinase 2 expression and hexokinase 2 silencing on glycolysis

**Figure 3:**
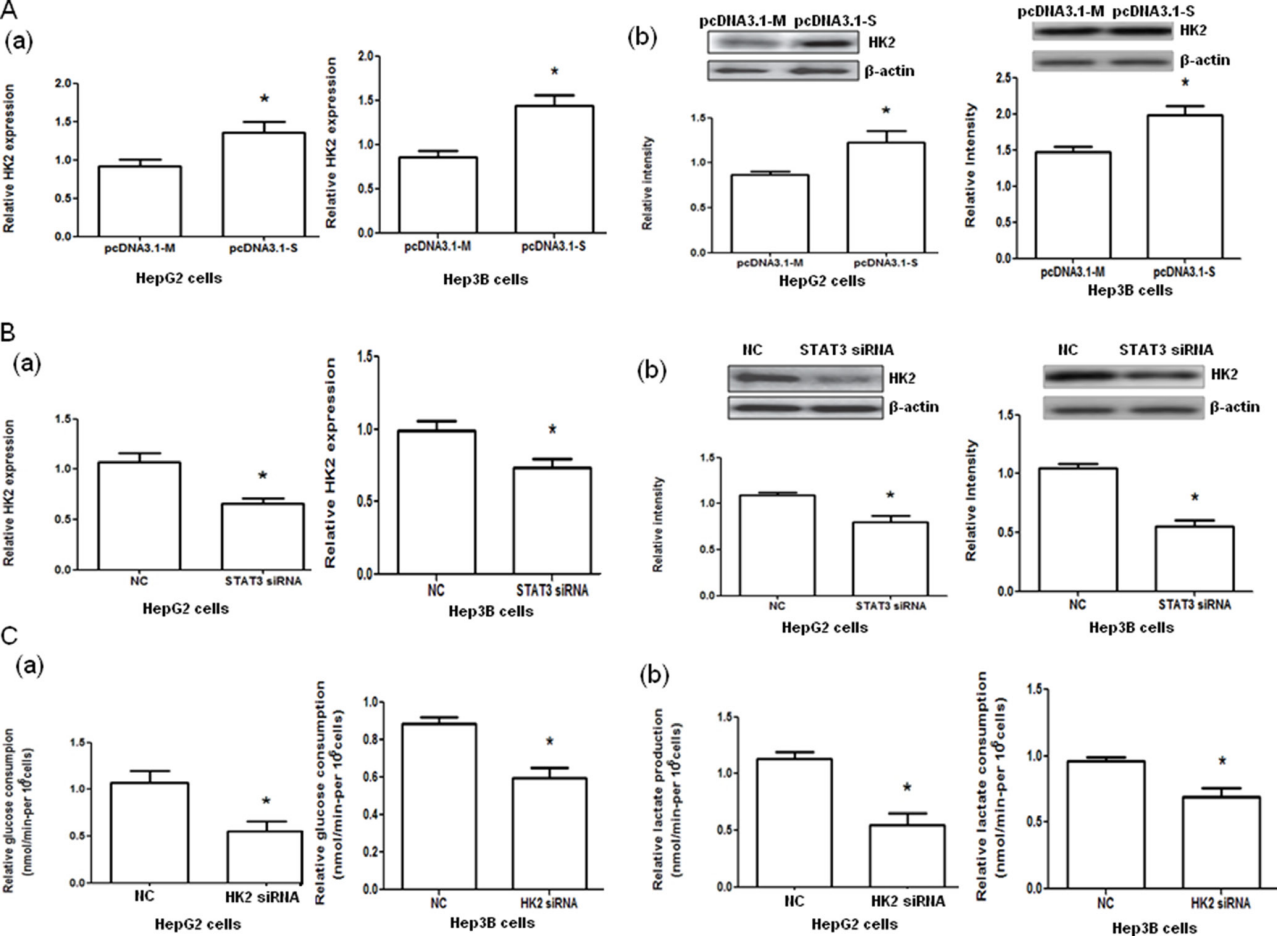
Hexokinase 2 (HK2) mRNA and protein expression in HepG2 and Hep3B cells with STAT3 overexpression and interference and glucose consumption and lactate production in HepG2 and Hep3B cells with HK2 interference (**A**) The HK2 mRNA (a) and protein (b) expression in HepG2 and Hep3B cells transfected with pcDNA3.1-Mock (pcDNA3.1-M) and pcDNA3.1-STAT3 (pcDNA3.1-S) determined by quantitative PCR and Western blot analysis, respectively. (**B**) The HK2 mRNA (a) and protein (b) expression in HepG2 and Hep3B cells transfected with negative control (NC) or STAT3 siRNA determined by quantitative PCR and Western blot analysis, respectively. (**C**) The glucose consumption (a) and lactate production (b) in HepG2 and Hep3B cells transfected with negative control (NC) RNA or HK2 siRNA. *P < 0.05.

HepG2 and Hep3B cells transfected with pcDNA3.1-STAT3 (pcDNA3.1-S) significantly increased HK2 mRNA and protein expressions (both *P <* 0.05, Figure 3A) compared with pcDNA3.1-Mock (pcDNA3.1-M). However, the transfection of HepG2 and Hep3B cells with STAT3 siRNA significantly decreased HK2 mRNA and protein expressions (both *P <* 0.05, Figure 3B). Furthermore, the glucose consumption and lactate production in HepG2 and Hep3B cells were significantly decreased when the cells were transfected with HK2 siRNA compared with those transfected with negative control RNA (both *P <* 0.05, Figure 3C, Supplementary Table 1).

### Effect of rapamycin on hexokinase 2 expression and glycolysis

**Figure 4:**
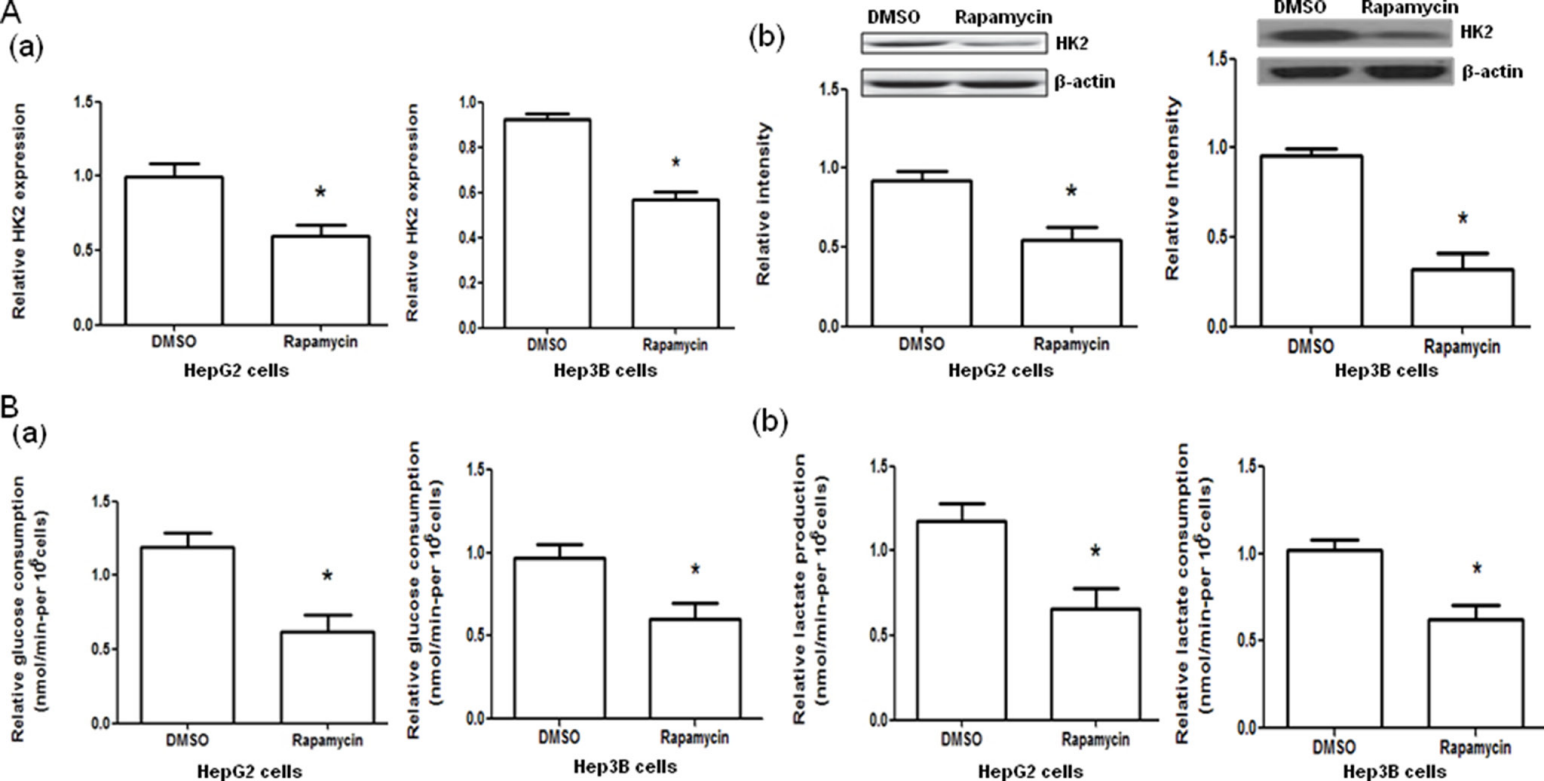
Effect of rapamycin on hexokinase 2 (HK2) mRNA and protein expression and glucose consumption and lactate production in HepG2 and Hep3B cells (**A**) The HK2 mRNA (a) and protein (b) expression in HepG2 and Hep3B cells treated with DMSO or rapamycin. (**B**) The glucose consumption (a) and lactate production (b) in HepG2 and Hep3B cells treated with DMSO or rapamycin. *P < 0.05.

To examine the effect of rapamycin on the glycolysis through mTOR-HK2 pathway in the cells used, treatment of HepG2 and Hep3B cells with rapamycin was performed. The treatment of HepG2 and Hep3B cells with rapamycin significantly decreased the HK2 mRNA and protein expression (both *P <* 0.05, Figure 4A) and the glucose consumption and lactate production (both *P <* 0.05, Figure 4B, Supplementary Table 1) compared with negative controls.

## DISCUSSION

STAT3 plays a critical role in tumor development, angiogenesis, and metastasis [25, 26]. It has also been shown that STAT3 promotes HCC initiation and malignant progression and blocking the activation of STAT3 inhibits the growth of human HCC [12, 27–30]. Reprogramming of metabolism involving an increase in glycolysis is a characteristic feature of HCC [23, 24]. Whether STAT3 may potentiate HCC energy metabolism remains largely unknown. To this end, we examined the effect of STAT3 on glucose metabolism in HepG2 and Hep3B cells. The results showed that overexpression of STAT3 in cells transfected with pcDNA3.1-S dramatically increased the glucose consumption and lactate production in HepG2 and Hep3B cells, while the downregulation of STAT3 by siRNA dramatically reduced the glucose consumption and lactate production, indicating that STAT3 enhances glycolysis in HCC cells.

“Warburg effect” is frequently observed in a variety of cancers [1–3]. HK2, the first rate-limiting enzyme of glycolysis, helps cancer cell with a highly glycolytic metabolism and promotes cancer cell proliferation [5–7]. HK2 is overexpressed in HCC [19] and has been documented as a pivotal player in the “Warburg effect” of HCC [21]. Studies have uncovered the potential role of STAT3 activation in glycolysis of cancer cells via HK2 pathway in breast cancer [17] and ovarian cancer cells [18]. Whether the role of HK2 in HCC is controlled by STAT3 has not been explored. Therefore, we examined the effects of STAT3 on the expression of HK2. Overexpression of STAT3 upregulated HK2 mRNA and protein expression, whereas silencing of STAT3 downregulated HK2 mRNA and protein expression. Furthermore, the silencing of HK2 is associated with reduced glucose consumption and lactate production. These results support that STAT3 regulates glycolysis through HK2 in HCC cells.

STAT3 is well known as a downstream factor of mTOR [31, 32] and rapamycin can down-regulate STAT3 expression through mTOR. The mTOR-STAT3 signaling pathway plays a crucial role in regulating cell growth, survival and metabolism [32–34]. Studies have reported that mTOR-STAT3 contributes to the process of proto-oncogenes and tumor suppressors by modulating the expression of various genes required for tumor cell survival, proliferation, angiogenesis, and metastasis [35, 36]. Our study indicated that STAT3 is a transcriptional activator for HK2. Rapamycin inhibited HK2 mRNA and protein expression. The glucose consumption and lactate production were dramatically reduced by rapamycin. These results suggest that rapamycin decreases glycolysis by mTOR-STAT3-HK2 pathway in HCC cells.

Rapamycin treatment, interference of STAT3 or inhibition of HK2, alone or in combination, has been indicated to be able to suppress the growth of HCC cells [23, 32, 36–40] and the invasion and metastasis behavior of HCC cells [30]. Our study provides evidence that STAT3 promotes HCC cell glycolysis metabolism via HK2 and the mTOR-STAT3-HK2 pathway is involved in the glycolysis of HCC cells, suggesting the potential by blocking HCC glycolysis through targeting multiple molecules of mTOR-STAT3-HK2 pathway in HCC treatment.

In summary, this study showed that STAT3 promotes glycolysis and regulates HK2 expression associated with the glycolysis of HepG2 and Hep3B cells. Interference of HK2 expression significantly reduces glycolysis of HepG2 and Hep3B cells. Rapamycin treatment of HepG2 and Hep3B cells decreases HK2 expression which is associated with reduced glycolysis. These findings suggest that the mTOR-STAT3-HK2 pathway is involved in the glycolysis of HCC and may thus provide potential multiple targets through interrupting glycolysis for HCC treatment.

## MATERIALS AND METHODS

### Plasmid constructs and siRNAs

Primers were synthesized by Sangon Biotech Co. Ltd ( Shanghai, Chnia) to amplify the whole DNA fragment of STAT3. The sequences were 5′-CCGGCTAGCATGGCCCAATGGAATCAG-3′, and 5′-CCCAAGCTTTCACATGGGGGAGGTAGC-3′ for forward primer and reverse primer, respectively. Recombinant plasmid pcDNA3.1(+)-EGFP-STAT3 and pcDNA3.1(+)-EGFP-Mock, designated as pcDNA3.1- STAT3 (pcDNA3.1-S) and pcDNA3.1-Mock (pcDNA3.1-M), respectively, were constructed by Integrated Medical Information (Xi`an, Shaanxi, China). The constructs were confirmed by DNA sequencing. The STAT3 siRNA and HK2 siRNA or the nonspecific negative control RNA were synthesized by Shanghai GeneChem Co., Ltd (Shanghai, China).

### Cell culture and transfection

The human hepatocellular carcinoma cell line HepG2 was obtained from Dr. Yawen Wang (Clinical Laboratory, The First Affiliated Hospital of Xi’an Jiaotong University, Xi’an, China), and the cell line Hep3B was purchased from Integrated Medical Information (Xi`an, Shaanxi, China). HepG2 cells and Hep3B cells were cultured in DMEM with 15% (for HepG2 cells) or 10% (for Hep3B cells) fetal bovine serum, 100 U/ml penicillin, and 100 μg/ml streptomycin at 37°C in a humidified atmosphere with 5% CO2.

pcDNA3.1-S or pcDNA3.1-M were transfected into cells with Lipofectamine 3000 (Invitrogen, Carlsbad, CA, USA).

Inhibition of the activity of mammalian target of rapamycin (mTOR), which is activated by STAT3, was performed by using rapamycin (Cell Signaling Technology, Danvers, MA, USA). Transfection of cells with STAT3 siRNA, HK2 siRNA or negative control RNA was carried out by using X-treme GENE siRNA Transfection Reagent (Roche, Nutley, NJ, USA) according to the manufactuter's instructions.

### Real-time quantitative PCR analysis

The total RNA was extracted from HepG2 or Hep3B cells by Trizol Reagent (TaKaRa Bio Inc., Dalian, China). RNA was subjected to reverse transcription reactions according to the manufactuter's instructions (Thermo Fisher Scientific Inc., Shanghai, China). RT-PCR was carried out with SYBR Premix Ex Taq II (Roche, Nutley, NJ, USA), and SLAN Real-time PCR System (Shanghai Hongshi Medical Technology Co., Ltd, Shanghai, China). Cycling conditions were as follows: 95°C for 10 min, then 40 cycles of 95°C for 10 sec, 60°C for 30 sec and 72°C for 30 sec and a final extension at 72°C for 2 min. The HK2 primers were: forward 5′-GCCTTTCCGTCCCAGCCTTTAGCC-3′, and reverse 5′-GGACTCCTGCGCCGGAGTTTCATG-3′. The STAT3 primers were: forward 5′-GAGAATCGTGGAGCTGT TTAG-3′, and reverse 5′-GACCAGCAACCTGACTT TAG-3′. The mRNA expression was normalized with GAPDH (forward 5′-GTATGACAACAGCCTCAAGA-3′, and reverse 5′-GTCCTTCCACGATACCAAAG-3′). All experiments were carried out in triplicate. The RT-PCR results were analyzed using the 2^−ΔΔCt^ method.

### Western blot analysis

Logarithmic growing cells were harvested for Western blotting 48 h after transfection. Proteins were probed with HK2, phospho-STAT3, or β-actin (Sigma-Aldrich Corporation, Shanghai, China) monoclonal antibody, and were assessed by BeyoECL Plus (Beyotime, Haimen, China). The films were scanned, and the density of the bands was quantified. Each experiment was performed in triplicate.

### Glucose consumption and lactate production assay

Glucose consumption and lactate production were analyzed as described previously [41]. The supernatants of cell culture media were collected. According to the manufactuter`s instructions, glucose levels and lactate levels were determined using a Glucose Assay kit and a Lactate Assay kit (Sigma-Aldrich Corporation, Shanghai, China), respectively.

### Statistical analysis

All data were analyzed using SPSS statistics 17.0 version (SPSS Inc., Chicago, IL, USA). All results were presented as mean ± S.D. Comparisons of the differences between groups relative to their paired controls, were evaluated by a Student's *t-test*. Statistical significance was expressed as a *P value*.

## SUPPLEMENTARY MATERIALS TABLES




