# Supplementary material for: STAT3 regulates glycolysis via targeting hexokinase 2 in hepatocellular carcinoma cells
Source: Oncotarget. 2017 Mar 1;8(15):24777–84. doi: 10.18632/oncotarget.15801 (PMC5421887; doi:10.18632/oncotarget.15801)
Supplement: Supplementary file 1 [file oncotarget-08-24777-s001.pdf]

STAT3 regulates glycolysis via targeting hexokinase 2 in hepatocellular carcinoma cells

Supplementary Materials

Supplementary Table 1: Supplementary Table 1. Original data of the experiments in HepG2 and Hep3B cells. See Supplementary_Table_1
