# Supplementary material for: STAT3 regulates glycolysis via targeting hexokinase 2 in hepatocellular carcinoma cells
Source: Oncotarget. 2017 Mar 1;8(15):24777–84. doi: 10.18632/oncotarget.15801 (PMC5421887; doi:10.18632/oncotarget.15801)
Supplement: Supplementary file 2 [file oncotarget-08-24777-s002.doc]

STAT3 regulates Glycolysis via Targeting Hexokinase 2 in Hepatocellular Carcinoma cells

**Supplementary Table 1.** Original data of the experiments in HepG2 and Hep3B cells

|  | | | | STAT3 mRNA expression in HepG2 and Hep3B cells transfected with pcDNA3.1-M or pcDNA3.1-S | | | | | Glucose consumption in HepG2 and Hep3B cells transfected with pcDNA3.1-M or pcDNA3.1-S | | | |
| --- | --- | --- | --- | --- | --- | --- | --- | --- | --- | --- | --- | --- |
|  |  |  |  | STAT3 expression | | | | | Glu consumption | | | |
|  |  |  |  | pcDNA3.1-M | | | pcDNA3.1-S | | pcDNA3.1-M | | pcDNA3.1-S | |
| HepG2 | | | | 8.9901 | | | 177.8093 | | 0.9334 | | 1.7890 | |
|  |  |  |  | 8.0211 | | | 184.2109 | | 1.0454 | | 2.2217 | |
|  |  |  |  | 9.1026 | | | 193.5321 | | 0.8976 | | 2.0314 | |
|  |  |  |  | *P*=0.0007 | | | | | *P*=0.0090 | | | |
| Hep3B | | | | 1.0001 | | | 379.50 | | 0.3127 | | 0.5867 | |
|  |  |  |  | 2.0010 | | | 343.20 | | 0.2815 | | 0.5467 | |
|  |  |  |  | 0.9114 | | | 400.10 | | 0.3559 | | 0.6510 | |
|  |  |  |  | *P*=0.0021 | | | | | *P*=0.0010 | | | |
|  | | | | Lactate production in HepG2 and Hep3B cells transfected with pcDNA3.1-M or pcDNA3.1-S | | | | | Glucose consumption in HepG2 and Hep3B cells transfected with negative control (NC) RNA or STAT3 siRNA. | | | |
|  |  |  |  | Lac production | | | | | Glu consumption | | | |
|  |  |  |  | pcDNA3.1-M | | | pcDNA3.1-S | | NC | | STAT3siRNA | |
| HepG2 | | | | 1.0331 | | | 1.8379 | | 0.9801 | | 0.3979 | |
|  |  |  |  | 0.9488 | | | 2.1533 | | 1.0047 | | 0.5524 | |
|  |  |  |  | 0.9143 | | | 1.9920 | | 0.8996 | | 0.5076 | |
|  |  |  |  | *P*=0.0129 | | | | | *P*=0.0136 | | | |
| Hep3B | | | | 0.3290 | | | 0.8355 | | 0.9732 | | 0.6086 | |
|  |  |  |  | 0.3951 | | | 0.8736 | | 0.9070 | | 0.5627 | |
|  |  |  |  | 0.2225 | | | 0.7643 | | 0.8434 | | 0.6346 | |
|  |  |  |  | *P*=0.0013 | | | | | *P*=0.0246 | | | |
|  | | | Lactate production in HepG2 and Hep3B cells transfected with negative control (NC) RNA or STAT3 siRNA. | | | | | |  | | | |
|  |  |  | Lac production | | | | | |  | | | |
|  |  |  | NC | | | | STAT3siRNA | |  | | | |
| HepG2 | | | 1.0018 | | | | 0.6144 | |  | | | |
|  |  |  | 0.9714 | | | | 0.5173 | |  | | | |
|  |  |  | 0.9211 | | | | 0.6386 | |  | | | |
|  |  |  | *P*=0.0173 | | | | | |  | | | |
| Hep3B | | | 0.9031 | | | | 0.5871 | |  | | | |
|  |  |  | 0.9586 | | | | 0.5901 | |  | | | |
|  |  |  | 0.8256 | | | | 0.4236 | |  | | | |
|  |  |  | *P*=0.0047 | | | | | |  | | | |
|  | | | | HK2 mRNA expression in HepG2 and Hep3B cells transfected with pcDNA3.1-M or pcDNA3.1-S | | | | | HK2 mRNA expression in HepG2 and Hep3B cells transfected with negative control (NC) or STAT3 siRNA | | | |
|  |  |  |  | HK2 expression | | | | | HK2 expression | | | |
|  |  |  |  | pcDNA3.1-M | | | pcDNA3.1-S | | NC | | STAT3siRNA | |
| HepG2 | | | | 0.8121 | | | 1.5543 | | 1.1203 | | 0.694 | |
|  |  |  |  | 0.9132 | | | 1.3421 | | 1.0931 | | 0.7427 | |
|  |  |  |  | 0.9579 | | | 1.3936 | | 0.9177 | | 0.5883 | |
|  |  |  |  | *P*=0.0353 | | | | | *P*=0.0063 | | | |
| Hep3B | | | | 0.7988 | | | 1.5530 | | 1.0174 | | 0.7914 | |
|  |  |  |  | 0.8665 | | | 1.3062 | | 0.9134 | | 0.6712 | |
|  |  |  |  | 0.9342 | | | 1.4533 | | 1.0384 | | 0.7521 | |
|  |  |  |  | *P*=0.0263 | | | | | *P*=0.0051 | | | |
|  | | | | Glucose consumption in HepG2 and Hep3B cells transfected with negative control (NC) RNA or HK2 siRNA. | | | | | Lactate production in HepG2 and Hep3B cells transfected with negative control (NC) RNA or HK2 siRNA. | | | |
|  |  |  |  | Glu consumption | | | | | Lac production | | | |
|  |  |  |  | NC | | | HK2 siRNA | | NC | | HK2 siRNA | |
| HepG2 | | | | 1.0179 | | | 0.4699 | | 1.0532 | | 0.4877 | |
|  |  |  |  | 1.1124 | | | 0.7124 | | 1.1321 | | 0.7314 | |
|  |  |  |  | 1.2503 | | | 0.6355 | | 1.0157 | | 0.5833 | |
|  |  |  |  | *P*=0.0145 | | | | | *P*=0.0115 | | | |
| Hep3B | | | | 0.9214 | | | 0.5668 | | 0.9931 | | 0.6811 | |
|  |  |  |  | 0.8816 | | | 0.5564 | | 0.9586 | | 0.7601 | |
|  |  |  |  | 0.8556 | | | 0.6609 | | 0.9256 | | 0.6232 | |
|  |  |  |  | *P*=0.0273 | | | | | *P*=0.0175 | | | |
|  | | | | HK2 mRNA expression in HepG2 and Hep3B cells treated with DMSO or rapamycin | | | | | Glucose consumption in HepG2 and Hep3B cells treated with DMSO or rapamycin | | | |
|  |  |  |  | HK2 expression | | | | | Glu consumption | | | |
|  |  |  |  | DMSO | | | Rapamycin | | DMSO | | Rapamycin | |
| HepG2 | | | | 0.9804 | | | 0.5025 | | 1.1806 | | 0.6806 | |
|  |  |  |  | 1.0547 | | | 0.4488 | | 1.0386 | | 0.5473 | |
|  |  |  |  | 0.9095 | | | 0.5626 | | 1.0567 | | 0.4980 | |
|  |  |  |  | *P*=0.0237 | | | | | *P*=0.0017 | | | |
| Hep3B | | | | 0.9733 | | | 0.5725 | | 0.8769 | | 0.6325 | |
|  |  |  |  | 0.8867 | | | 0.6277 | | 0.9875 | | 0.4877 | |
|  |  |  |  | 0.9154 | | | 0.4976 | | 1.0432 | | 0.6764 | |
|  |  |  |  | *P*=0.0191 | | | | | *P*=0.0374 | | | |
|  | | | Lactate production in HepG2 and Hep3B cells treated with DMSO or rapamycin | | | | | |  | | | |
|  |  |  | Lac production | | | | | |  | | | |
|  |  |  | DMSO | | | Rapamycin | | |  | | | |
| HepG2 | | | 1.1833 | | | 0.6974 | | |  | | | |
|  |  |  | 1.0867 | | | 0.4823 | | |  | | | |
|  |  |  | 1.0054 | | | 0.5781 | | |  | | | |
|  |  |  | *P*=0.0104 | | | | | |  | | | |
| Hep3B | | | 0.9748 | | | 0.6248 | | |  | | | |
|  |  |  | 1.0864 | | | 0.5327 | | |  | | | |
|  |  |  | 0.9965 | | | 0.7039 | | |  | | | |
|  |  |  | *P*=0.0373 | | | | | |  | | | |
|  | | | | | STAT3 protein expression in HepG2 and Hep3B cells transfected with pcDNA3.1-M or pcDNA3.1-S | | | | | HK2 protein expression in HepG2 and Hep3B cells transfected withpcDNA3.1-M or pcDNA3.1-S | | |
|  |  |  |  |  | STAT3 expression | | | | | HK2 expression | | |
|  |  |  |  |  | pcDNA3.1-M | | | pcDNA3.1-S | | pcDNA3.1-M | | pcDNA3.1-S |
| HepG2 | | Western blot | | | 0.5462 | | | 1.7732 | | 0.8372 | | 1.3322 |
|  |  |  |  |  | 0.6875 | | | 1.5341 | | 0.9051 | | 1.1315 |
|  |  |  |  |  | 0.4988 | | | 1.4707 | | 0.7988 | | 1.2637 |
|  |  |  |  |  | *P*=0.0119 | | | | | *P*=0.0432 | | |
| Hep3B | Western blot | | | | 1.2945 | | | 1.7436 | | 1.6283 | | 2.1342 |
|  |  |  |  |  | 1.5673 | | | 1.9726 | | 1.4074 | | 1.7264 |
|  |  |  |  |  | 1.4433 | | | 2.1427 | | 1.3866 | | 2.0936 |
|  |  |  |  |  | *P*=0.0299 | | | | | *P*=0.0449 | | |
|  | | | | | HK2 protein expression in HepG2 and Hep3B cells transfected with negative control (NC) RNA or STAT3 siRNA | | | | | HK2 protein expression in HepG2 and Hep3B cells treated with DMSO or rapamycin | | |
|  |  |  |  |  | HK2 expression | | | | | HK2 expression | | |
|  |  |  |  |  | NC | | | STAT3siRNA | | DMSO | | Rapamycin |
| HepG2 | | Western blot | | | 0.9873 | | | 0.7432 | | 0.8743 | | 0.5487 |
|  |  |  |  |  | 1.0421 | | | 0.8065 | | 0.8899 | | 0.4269 |
|  |  |  |  |  | 1.0076 | | | 0.6244 | | 0.9474 | | 0.5272 |
|  |  |  |  |  | *P*=0.0266 | | | | | *P*=0.0100 | | |
| Hep3B | Western blot | | | | 1.1086 | | | 0.4770 | | 1.0177 | | 0.1381 |
|  |  |  |  |  | 1.0474 | | | 0.5273 | | 0.9657 | | 0.3756 |
|  |  |  |  |  | 0.9954 | | | 0.6534 | | 0.8974 | | 0.4423 |
|  |  |  |  |  | *P*=0.0275 | | | | | *P*=0.0360 | | |
